# Comparison of 68Ga-DOTANOC with 18F-FDG using PET/MRI imaging in patients with pulmonary tuberculosis

**DOI:** 10.1038/s41598-020-71127-2

**Published:** 2020-08-28

**Authors:** Claire M. Naftalin, Francesca Leek, James T. P. D. Hallinan, Lih Kin Khor, John J. Totman, Jing Wang, Yee Tang Wang, Nicholas I. Paton

**Affiliations:** 1 Department of Medicine, Yong Loo Lin School of Medicine, National University of Singapore, 10 Medical Drive, Singapore, 117597 Singapore; 2 Clinical Imaging Research Centre, National University of Singapore, Singapore, Singapore; 3 Department of Diagnostic Imaging, National University Health System, Singapore, Singapore; 4 Tuberculosis Control Unit, Tan Tock Seng Hospital, Singapore, Singapore; 5 University Medicine Cluster, National University Health System, Singapore, Singapore

**Keywords:** Infectious diseases, Tuberculosis, Biomarkers

## Abstract

We compared the somatostatin analog radioligand, DOTANOC, with FDG, to determine whether there was increased detection of active or sub-clinical lesions in pulmonary tuberculosis (TB) with DOTANOC. Three groups were recruited: (1) active pulmonary TB; (2) IGRA-positive household TB contacts; (3) pneumonia (non-TB). DOTANOC PET/MRI followed by FDG PET/MRI was performed in active TB and pneumonia groups. TB contacts underwent FDG PET/MRI, then DOTANOC PET/MRI if abnormalities were detected. Quantitative and qualitative analyses were performed for total lung and individual lesions. Eight active TB participants, three TB contacts and three pneumonia patients had paired PET/MRI scans. In the active TB group, median SUVmax_[FDG]_ for parenchymal lesions was 7.69 (range 3.00–15.88); median SUVmax_[DOTANOC]_ was 2.59 (1.48–6.40). Regions of tracer uptake were fairly similar for both radioligands, albeit more diffusely distributed in the FDG scans. In TB contacts, two PET/MRIs had parenchymal lesions detected with FDG (SUVmax 5.50 and 1.82), with corresponding DOTANOC uptake < 1. FDG and DOTANOC uptake was similar in pneumonia patients (SUVmax_[FDG]_ 4.17–6.18; SUVmax_[DOTANOC]_ 2.92–4.78). DOTANOC can detect pulmonary TB lesions, but FDG is more sensitive for both active and sub-clinical lesions. FDG remains the preferred ligand for clinical studies, although DOTANOC may provide additional value for pathogenesis studies.

## Introduction

Tuberculosis (TB) disease affects 10 million people worldwide every year, and is the leading cause of death from an infectious disease^1^. New TB biomarkers are required for a variety of applications, including detection of sub-clinical disease for early intervention to prevent disease progression; detection of new active TB cases; and for monitoring of treatment response in clinical practice or in clinical trials of new TB therapies.

Positron emission tomography (PET) imaging in combination with structural imaging may have value as a biomarker for detecting subclinical TB disease, active disease where the diagnosis is problematical, and an outcome measure in clinical trials^2–7^. The standard PET ligand, [18F]fluoro-2-deoxy-2-d-glucose (FDG), accumulates in cells with high levels of glucose metabolism, and is a non-specific marker of inflammation. There may be additional value in using alternatives to FDG, if these have higher sensitivity or specificity for TB-infected cells^8^.

Somatostatin analog PET radiotracers are useful in evaluating neuro-endocrine tumours^9–11^ and other cancers' cells^12–15^ that have upregulated cell surface somatostatin receptors (SSTRs). These receptors are also overexpressed on activated macrophages^16,17^, which are a key cell population infected by TB, residing within granulomas^18,19^. Over-expression of somatostatin receptors in granulomas within lymph nodes of TB patients have been visualised using in vitro autoradiography^20^, and SSTR-positive cells have been detected using immunohistochemistry analysis in granulomas from multiple granulomatous conditions^21,22^ including pulmonary nodules in TB^23^.

In this study we compared PET in combination with magnetic resonance imaging (MRI) using the radiolabeled somatostatin analog 68Ga-DOTA-1-NaI3-octreotide (DOTANOC) with FDG to determine whether DOTANOC might increase the detection of active and sub-clinical TB lesions.

## Results

### Participants

Eight participants with active TB, three TB contacts and three pneumonia patients underwent paired FDG and DOTANOC PET/MRI scans. One additional participant with active TB was enrolled, but PET imaging data was incomplete; seventeen additional contacts were enrolled but did not have lesions visible on the FDG scan and did not proceed to DOTANOC scanning. These participants are not reported further.

In the active TB group, there were six males, two females; median age of 37.5 years old (age range 22–61); five Chinese, two Malay, one Burmese. All patients had culture-confirmed drug-sensitive pulmonary TB (1 smear negative, 2 smear 2+, 2 smear 3+, 3 smear 4+). The median number of days on anti-tuberculous therapy at first scan was 18 days (range 8–29); all participants were taking standard combination therapy (rifampicin, isoniazid, ethambutol, pyrazinamide).

The three TB contacts were all female, aged 29–51 years old. Two participants were taking isoniazid TB prophylaxis (for 3 and 5 days prior to FDG scan). The three participants in the pneumonia group were male, aged between 36 and 73 years old. Diagnosis was clinical; patients had been taking antibiotic treatment for 2, 5 and 6 days at the time of the FDG scan with a rapid response to antibiotic therapy. All participants were HIV negative.

### DOTANOC PET/MRI and FDG PET/MRI analysis

**Figure 1:**
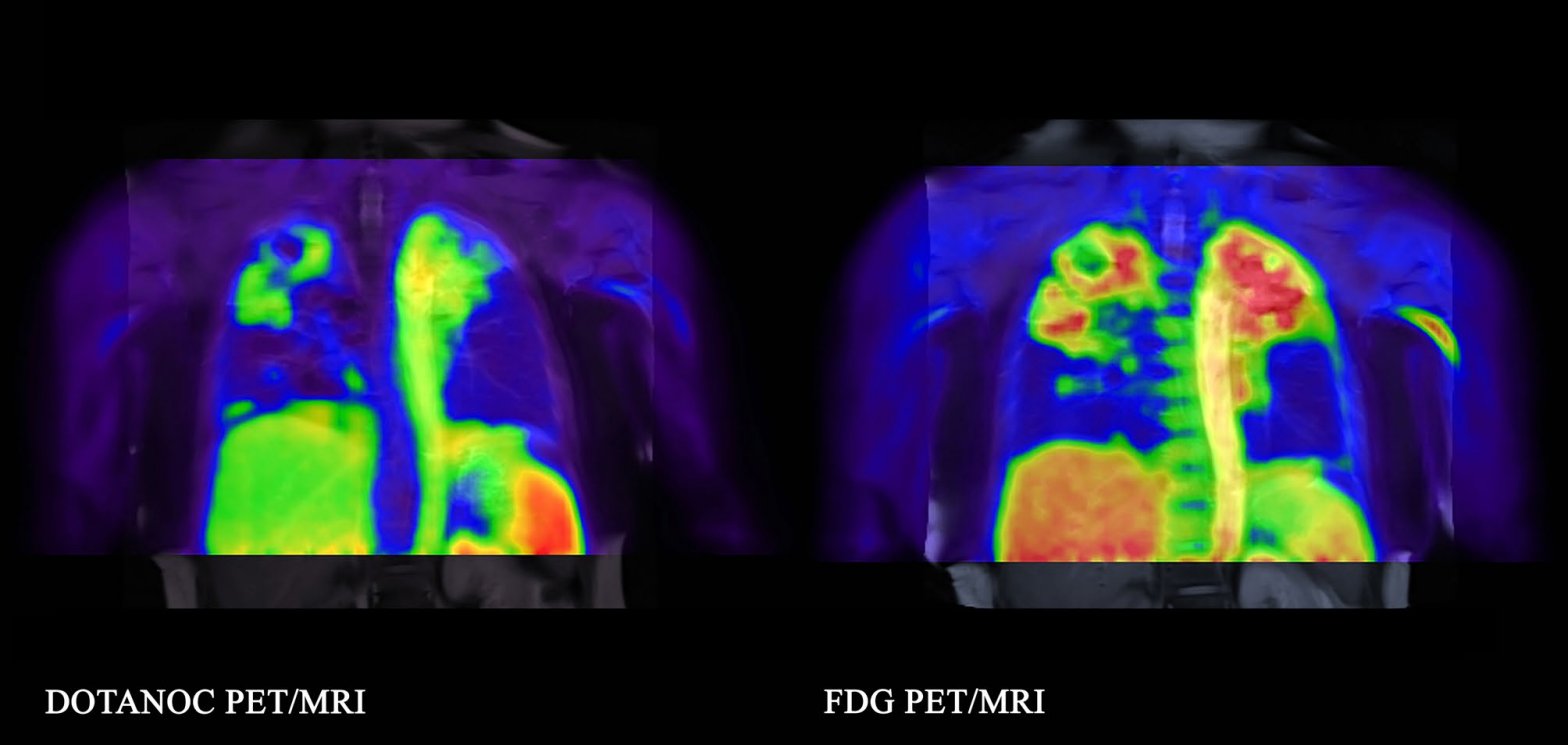
Example of diffuse uptake of FDG (SUVmax 8.22) compared to DOTANOC uptake (SUVmax 2.96) which is more discrete. 55-year-old woman with pulmonary TB, smear 3+, on Day 8 of TB treatment.

**Figure 2:**
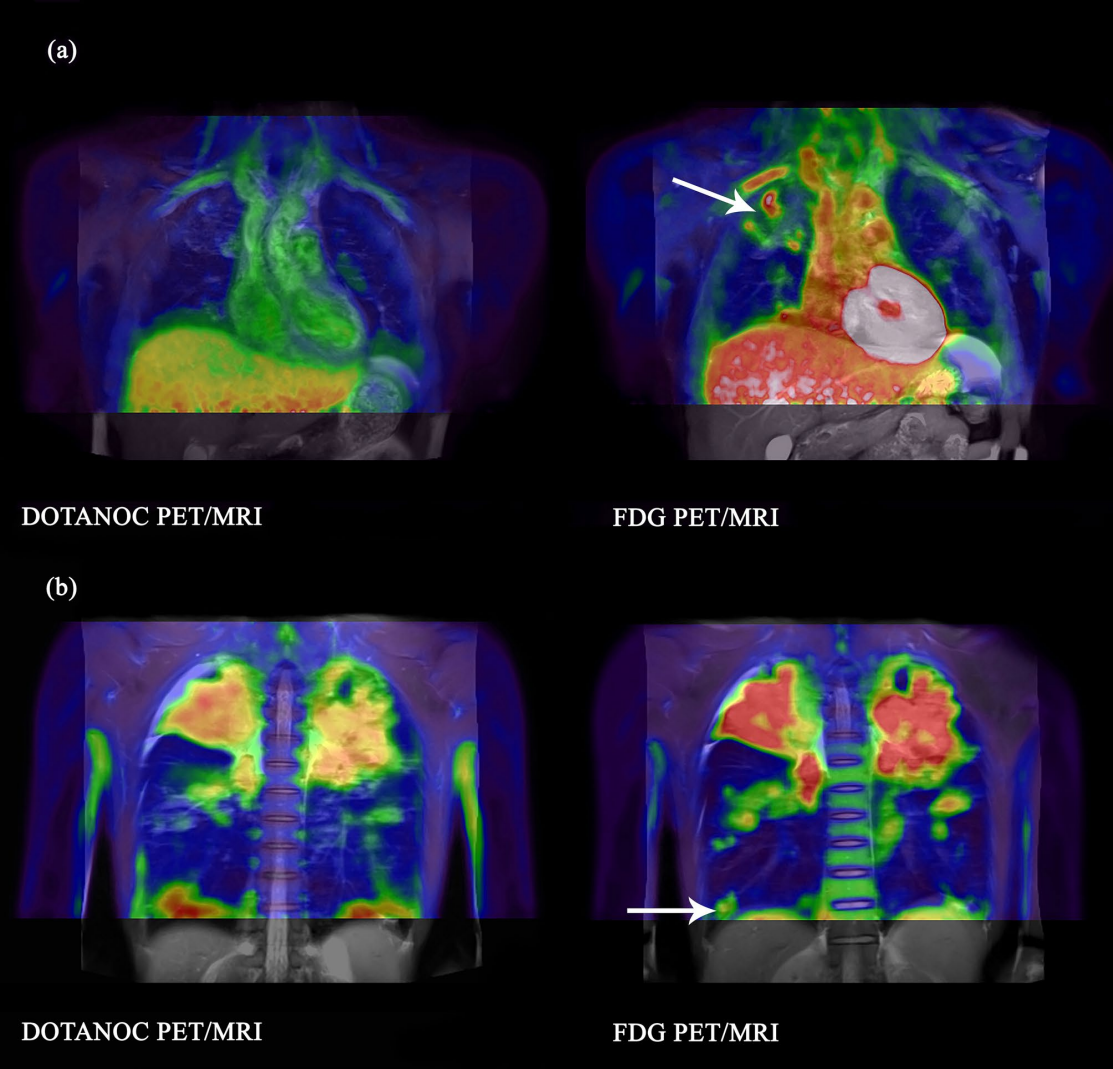
Lesions visualised with FDG but not DOTANOC in pulmonary TB (**a**) Lesions in right upper lobe visualised with FDG, largest volume 16.5 cm^3^, SUVmax 2.89. No uptake with DOTANOC. 22-year-old male, smear 2+, on TB treatment for 28 days. (**b**) Lesion in posterior basal segment of right lower lobe visualised with FDG, volume 4.3 cm^3^, SUVmax 2.50. No uptake with DOTANOC. 29-year-old male, smear 3+, on TB treatment for 25 days.

**Figure 3:**
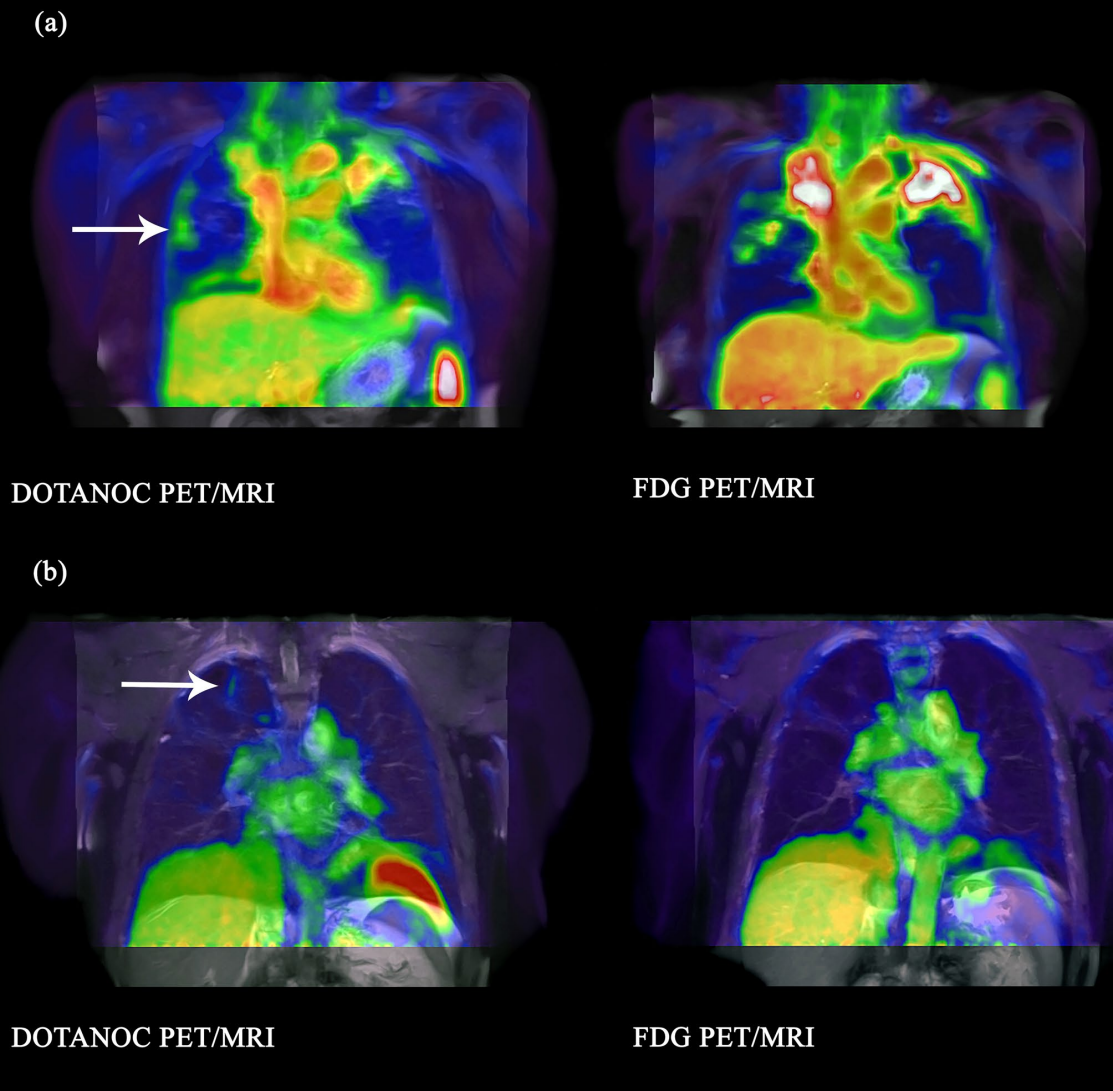
Lesions visualised with DOTANOC but not FDG in pulmonary TB (**a**) Lesion in right middle lobe visualised with DOTANOC, volume 0.6 cm^3^, SUVmax 1.92. No uptake with FDG. 55-year-old woman, smear 3+, on Day 8 of TB treatment. (**b**) Lesion at apex right upper lobe visualised with DOTANOC, volume 0.4 cm^3^, SUVmax 1.80. No uptake with FDG. 57-year-old man, smear negative, on Day 11 of TB treatment.

In the active TB group, the median total percentage of disease-affected lung was 10.8% (range 1.6–30.0%) with FDG and 10.8% (range 1.6–30.6%) with DOTANOC. The median maximum standardized uptake value (SUVmax) of diseased lung parenchyma was 7.69 (range 3.00–15.88) with FDG and 2.59 (range 1.48–6.40) with DOTANOC; median total lung SUVmax_[FDG]_:SUVmax_[DOTANOC]_ ratio was 2.60 (range 1.62–3.17). There was no significant correlation between total lung FDG:DOTANOC uptake ratio and time on TB treatment (rho = 0.14, p = 0.73). The distribution of disease was similar between FDG and DOTANOC scans but, in general, lesions appeared to be more diffuse with FDG, with clearer separation between adjacent lesions with DOTANOC (example in Fig. 1). A total of 33 individual lesions were identified in the eight patients (range 2–7 lesions per patient). Of the 22 lesions with both FDG and DOTANOC uptake, median individual lesion SUVmax _[FDG]_:SUVmax_[DOTANOC]_ ratio was 2.23 (range 0.87–3.90). Nine parenchymal lesions (in five scans, range 1–3 lesions per scan) had FDG uptake (median SUVmax 2.15, range 1.64–2.94; volume 4.3 cm^3^, range 0.6–16.5 cm^3^) but no DOTANOC uptake (examples in Fig. 2). Conversely, there were only two parenchymal lesions (one in each of two patients) that had DOTANOC uptake (SUVmax 1.80 and 1.92; volume 0.4 cm^3^ and 0.6 cm^3^) but no uptake with FDG (Fig. 3). One additional lesion in a diffuse area adjacent to scar tissue had FDG uptake (FDG SUVmax 2.62; volume 43.4 cm^3^) but no DOTANOC uptake. Active hilar and/or mediastinal lymph nodes were seen in four scans, visible both with FDG (SUVmax range 1.20–2.75) and DOTANOC (SUVmax range 0.95–2.53).

**Figure 4:**
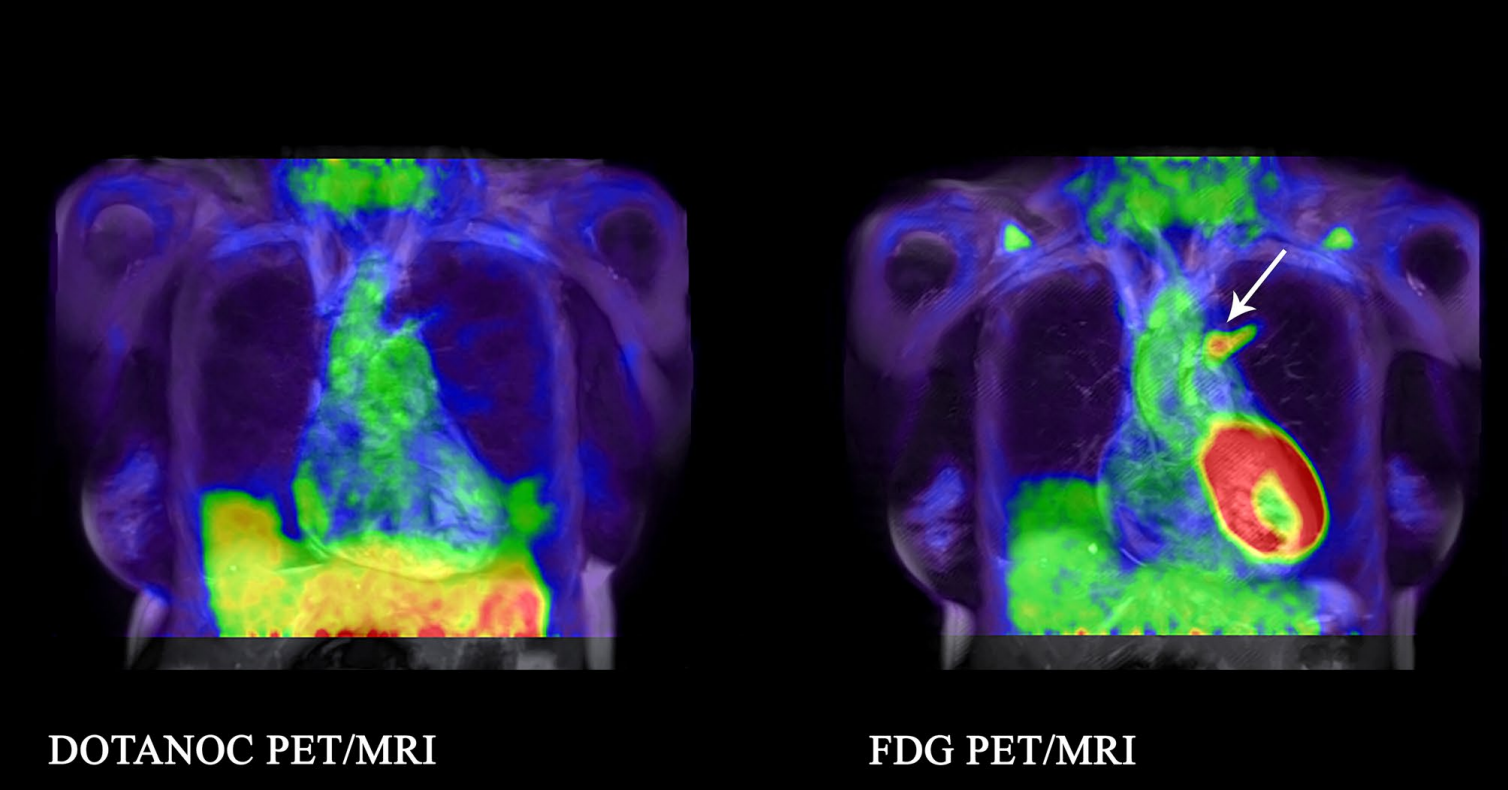
Lesion visualised in anterior segment of the left upper lobe adjacent to the aorta with FDG in 47-year-old female, IGRA-positive TB contact, taking isoniazid for 5 days. FDG SUVmax 5.50. No DOTANOC uptake.

In the TB contacts, two FDG scans had parenchymal lesions: one in the anterior segment of the left upper lobe (SUVmax 5.50; volume 5.2 cm^3^; Fig. 4) and one in the anterior segment of the right upper lobe (FDG SUVmax 1.82; volume 0.7 cm^3^); the corresponding DOTANOC scans both had SUVmax < 1. Two FDG scans showed increased uptake in lymph nodes, one with SUVmax 8.12 (DOTANOC scan, SUVmax 1.25); and one with SUVmax 2.72 (no uptake on DOTANOC scan). The other two DOTANOC scans did not show any increased uptake in lymph nodes.

**Figure 5:**
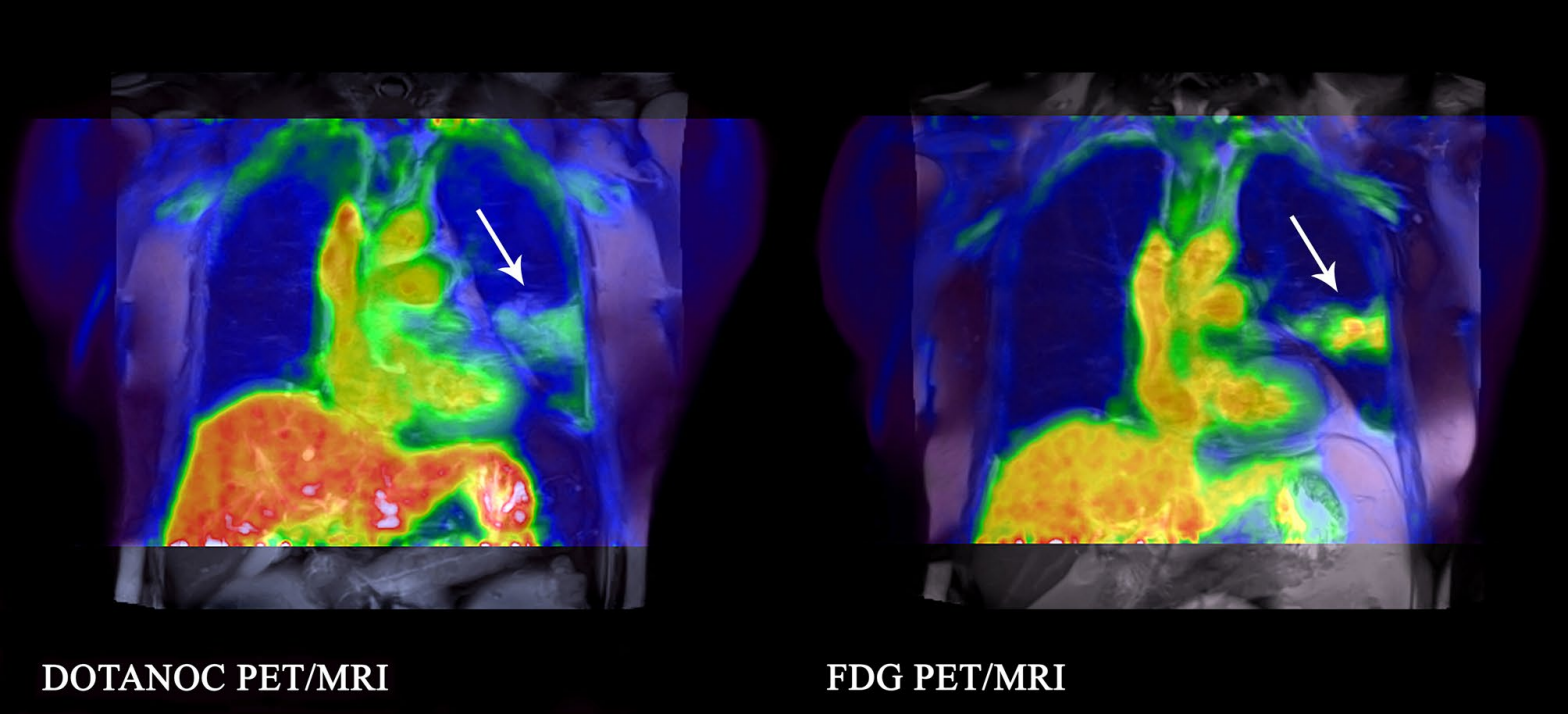
Similar distribution of tracer uptake of DOTANOC and FDG in a 73-year old male patient with non-TB pneumonia. Lesion visualised in the left lower lobe mainly involving the superior segment.

In the patients with pneumonia, large confluent areas of uptake were seen in all three scans with both FDG (SUVmax 4.17–6.18), and DOTANOC (SUVmax 2.92–4.78), with similar distribution in the two scans (example in Fig. 5). Two FDG scans showed increased uptake in lymph nodes, one with SUVmax 3.96 (no uptake on DOTANOC scan) and one with SUVmax 2.07 (DOTANOC SUVmax 1.27).

## Discussion

Our study shows that PET scanning using a somatostatin analogue radioligand can detect pulmonary lesions in patients with active TB. This finding is consistent with a recent case report showing intense DOTANOC uptake in a single mesenteric tuberculous lesion^24^, and several previous nuclear imaging studies using 99mTc- and 111In-labelled somatostatin analogs, that have shown increased uptake in pathological lesions in TB and other granulomatous diseases^20,25–27^. However, although we demonstrated that DOTANOC images TB lung disease, there does not appear to be any particular advantage over the standard ligand: the few discrepancies we observed in lesions visualised with the two ligands generally favoured FDG, and lesion uptake was, on average, more than two-fold higher with FDG for individual lesions and for total lung lesions. The two individual lesions visualised with DOTANOC, but not with FDG, were of low intensity and small volume.

The differences in lesion avidity we observed likely arise because the radioligands are detecting different aspects of TB-related pathology. Granulomas are rich in activated macrophages, but they also contain numerous other cell types including neutrophils, B-cells and T-cells. Furthermore, the cellular composition of individual granulomas varies, even within the same patient, and may evolve independently over time on TB therapy^28,29^. FDG measures glucose uptake and labels a broad range of metabolically-active cells including neutrophils, the predominant cell type infected with replicating *Mycobacterium tuberculosis* during active pulmonary TB^30^, and are present in lung tissue surrounding the granulomas^31^. In contrast, DOTANOC measures somatostatin receptors which are upregulated on activated macrophages in granulomas, as well as fibroblasts^32,33^, and B- and T-cells^34^; but not neutrophils^35^. The small number of parenchymal lesions visualised more intensely with DOTANOC may have had greater density of macrophages and other somatostatin receptor cell types, but with lower metabolic activity, than the predominantly FDG-avid parenchymal lesions. The greater diversity of cell types that FDG labels may explain the relatively diffuse FDG uptake contrasting with more discrete lesions seen with DOTANOC. The greater avidity of lesions with FDG than DOTANOC is consistent with a study in a TB macaque model comparing FDG with 64Cu-LLP2A^28^, a ligand that binds to very late antigen-4 (VLA-4), expressed especially on epithelioid macrophages and T cells in granulomas. Lesion avidity was significantly higher with FDG than 64Cu-LLP2A, particularly in early infection. The higher avidity seen with FDG than the comparator cell receptor-specific ligand observed both in that study and our study could be due to differences in the cell types labelled within lesions, differences in ligand penetration into the lesions or, most likely, the large increase in glycolytic metabolism in the anaerobic centre of granulomas (labelled with FDG, but not DOTANOC)^28^.

In the macaque study, the difference between FDG and receptor-specific ligand was most evident within 9 weeks of infection but diminished at later follow-up. In our study, considerations of radiation dose and patient acceptability limited us to performing one scan with each ligand, at a single time point. We studied TB patients relatively early during the course of treatment (within the first month) and it is possible that the relative lesion avidity detected with FDG and DOTANOC could change within each patient as treatment progresses. However, we also performed PET scans in household TB contacts, to determine whether DOTANOC could detect early disease following TB exposure, as we had identified using FDG in an earlier study^36^. The difference between FDG and DOTANOC in TB contacts was more marked than for patients with active TB: the parenchymal lesions seen on FDG were not visible with DOTANOC, and lymph node uptake on FDG was only visible in one of two DOTANOC scans. We cannot be certain whether the abnormalities seen in these contacts represent an early stage of active TB infection or whether they simply represent transient immune activity occurring early in infection^36^. The finding of more prominent differences between ligands in early disease is again keeping with that seen in macaques^28^. We included a group of patients with non-TB pneumonia as controls, to examine whether DOTANOC uptake might be specific for TB and therefore of potential diagnostic value in difficult-to-diagnose cases. However, we found clear uptake of DOTANOC in these pneumonia patients, with lesions similar in distribution to FDG, with only moderately reduced avidity. This is consistent with a few previous case reports of pneumonia detected as an incidental finding with SSTR scintigraphy^37–39^, although not in all reports^40^. In the case of non-TB pneumonia, DOTANOC may be labelling fibroblasts^32,33^, B- and T-cell lymphocytes^34^ and macrophages recruited to the lung in the later stages of pneumonia and which may express SSTRs^41^. Although other non-mycobacterial causes of granulomatous pneumonia such as sarcoidosis and fungal infections were not ruled out, we can be confident that this was not TB (based on rapid resolution without TB treatment) and we do not need to know the precise identification of the pathogen to support the conclusion that DOTANOC is not specific for TB and has limited value for diagnostic purposes.

The main limitation of this study is the small sample size, although it was adequate for the intended descriptive analysis. In patients with active TB, the DOTANOC scans were always performed 1–2 days after the FDG scan, but this delay could not explain the marked difference in uptake, given that abnormalities on PET scans of patients with TB are known to resolve only slowly on treatment, with residual activity detected even at or close to the end of treatment^5^. In the contacts group, DOTANOC PET/MRI was only performed for TB contacts with positive findings on the FDG scan (to minimise radiation exposure), so we cannot confidently rule out that DOTANOC may have detected lesions that FDG did not identify. However, this is unlikely given that DOTANOC appeared to be less sensitive than FDG for detecting individual lesions in active TB, or in the paired scans performed in contacts.

Our findings have a number of implications for future research. The lower avidity of lesions with DOTANOC suggest PET imaging using FDG is more suitable as an outcome parameter in TB clinical trials: the lower amplitude of measurement using DOTANOC might reduce power to detect differences between treatment groups over time. Furthermore, FDG is manufactured more widely at lower cost and has lower radiation dose, and is therefore of more practical utility. Our findings that DOTANOC has substantial uptake in non-TB pneumonia also reduces any value in the differential diagnosis of lung lesions. However, our findings suggest there may be further research value in longitudinal studies with paired PET ligands labelling different cellular populations or metabolic processes. Changes in the relative uptake of ligands may provide important insights into the mechanism of action of drugs or the host response in controlling TB. Further studies using radioligands labelling specific components of the mycobacteria such as Trehalose analogues^42^, or targets of specific metabolism pathways such as lipogenesis^43^, to differentiate between TB and other pathologies, may be a more productive direction for PET-based imaging research in TB.

In conclusion, we compared the somatostatin analog radioligand, DOTANOC, with the standard tracer, FDG, in detection of active pulmonary TB and sub-clinical pulmonary lesions. FDG was more sensitive than DOTANOC in detecting pulmonary TB lesions in both active and sub-clinical disease. DOTANOC uptake was not specific to TB lesions, showing comparable uptake to FDG in non-TB pneumonia. Future studies may identify promising TB imaging biomarkers by focusing on radioligands labelling specific targets of the mycobacteria, or pathways of TB metabolism.

## Methods

We studied three groups of participants. The active TB group comprised adults with pulmonary TB (diagnosis based on compatible chest X-ray (CXR) findings with a positive acid-fast bacilli smear or positive GeneXpert test or TB-culture positive), who had started TB therapy within the previous one month. The TB contact group comprised close household contacts (sleeping in same house for ≥ 1 month before TB patient started therapy) of a smear-positive TB patient diagnosed within the last 2 months; were Interferon-Gamma Release Assay (IGRA) positive; had never received treatment for active TB disease; and had no clinical or X-ray evidence of pulmonary TB. The pneumonia group comprised adults with a clinical diagnosis of bacterial pneumonia with compatible CXR findings, who had shown a clinical response to antibiotic treatment started within the previous 7 days. Common exclusion criteria for all groups included poorly-controlled diabetes, chronic kidney disease, malignancy requiring chemotherapy or radiotherapy, contraindication to radiation or MRI scanning, pregnancy or breast-feeding.

Participants had an HIV test and a standard posteroanterior CXR. The active TB group and pneumonia group underwent DOTANOC PET/MRI followed by FDG PET/MRI either 1 or 2 days later. The TB contact group underwent an initial FDG PET/MRI and if abnormalities were detected, this was followed by a DOTANOC PET/MRI between 1 and 7 days later.

The research received ethics approval from the National Healthcare Group’s Domain Specific Review Board in Singapore and regulatory approval was obtained from the Health Sciences Authority (HSA), Singapore. All research was performed in accordance with the relevant guidelines and regulations, and all participants provided written informed consent.

### PET/MRI imaging protocol

All PET/MRI scans were performed at the National University of Singapore (NUS) Clinical Imaging Research Centre using a Siemens Biograph mMR PET/MR scanner (Siemens Healthcare, Erlangen, Germany). Prior to FDG PET/MRI, participants fasted for 6 h, following which an intravenous injection of 18F-FDG (mean activity 138.4 ± 9.4 MBq) was given to each participant. Fasting was not required prior to DOTANOC PET/MRI. An intravenous injection of 68Ga-DOTANOC (191.7 ± 9.3 MBq) was given. Scans for both radioligands were commenced immediately and data acquired up to 80 min post injection.

The PET images were reconstructed using Ordinary-Poisson Ordered-Subset Expectation–Maximisation (OP-OSEM) with 3 iterations and 21 subsets. A Gaussian post-smoothing filter of 6 mm full-width at half maximum (FWHM) was applied. The matrix size was 172 × 172, with a voxel size of 4.17 × 4.17 mm and slice thickness of 2.03 mm.

The MRI data was acquired using 12-channel body coils. Dixon images were collected for the purpose of MR-based Attenuation Correction (MRAC).

### Imaging analysis

PET/MRI scans were evaluated and analysed by two independent radiologists; quantitative data was averaged, and any major discrepancies were agreed by consensus. Detailed analysis of each lesion was performed by a medical physicist. All evaluations were done blinded to clinical characteristics of the participants.

PET image analysis was performed on the reconstructed data acquired 60–70 min post injection, for both radioligands. Lung volumes were automatically segmented using ITK-SNAP^44^ on the MR navigated three-dimensional sampling perfection with application optimized contrasts using different flip-angle evolutions (3D-SPACE) image acquired at the participant’s first visit. The volumes of interest (VOI) were automatically propagated to the PET images^45,46^ and manually refined.

Any non-physiological uptake of FDG or DOTANOC in the lung above the mediastinal blood pool was considered abnormal^5,47,48^. All voxels contained within the VOI with radioligand uptake above mediastinal blood pool were automatically thresholded using commercially available software (PMOD). The SUV for the mediastinal blood pool was obtained by positioning a 1 cm spherical VOI in the descending aorta. VOIs were removed from analysis upon consensus that increased uptake was due to artefact or vasculature.

Evaluation of scans was standardised using a structured case report form including quantitative measures and specific qualitative characteristics of tracer uptake within each lung.

This was a pilot study and sample size was determined pragmatically according to feasible numbers to allow descriptive comparison of imaging findings between the ligands in the three clinical populations.

## Data Availability

The datasets generated and/or analysed during the current study are available from the corresponding author on reasonable request.
